# Sleep-related benefits to transitive inference are modulated by encoding strength and joint rank

**DOI:** 10.1101/lm.053787.123

**Published:** 2023-09

**Authors:** Tamas Foldes, Lorena Santamaria, Penny Lewis

**Affiliations:** Cardiff University Brain Research Imaging Centre (CUBRIC), Cardiff University, Cardiff, Wales CF24 4HQ, United Kingdom

## Abstract

Transitive inference is a measure of relational learning that has been shown to improve across sleep. Here, we examine this phenomenon further by studying the impact of encoding strength and joint rank. In experiment 1, participants learned adjacent premise pairs and were then tested on inferential problems derived from those pairs. In line with prior work, we found improved transitive inference performance after retention across a night of sleep compared with wake alone. Experiment 2 extended these findings using a within-subject design and found superior transitive inference performance on a hierarchy, consolidated across 27 h including sleep compared with just 3 h of wake. In both experiments, consolidation-related improvement was enhanced when presleep learning (i.e., encoding strength) was stronger. We also explored the interaction of these effects with the joint rank effect, in which items were scored according to their rank in the hierarchy, with more dominant item pairs having the lowest scores. Interestingly, the consolidation-related benefit was greatest for more dominant inference pairs (i.e., those with low joint rank scores). Overall, our findings provide further support for the improvement of transitive inference across a consolidation period that includes sleep. We additionally show that encoding strength and joint rank strongly modulate this effect.

Relational reasoning is the cognitive process of identifying and understanding relationships between stimuli or concepts. It involves the ability to identify patterns (for example, similarities and differences between stimuli) and to understand how they relate to each other (Halford et al. 2010). Relational reasoning is important for a wide range of cognitive tasks, such as problem-solving (Dumas et al. 2014), decision-making (Dumas 2016), and language acquisition (Gentner and Namy 2006). Transitive inference is a specific type of relational reasoning that involves making inferences about the relationships between items in a hierarchy. For example, if A is dominant to B, and B is dominant to C, then we can infer that A dominates C when probed (A?C). This type of inference requires the ability to reason about the relationships between stimuli based on their relative positions in a hierarchy. Transitivity, a property of all ordered sets, has been studied extensively in human developmental psychology for over a century (Piaget 1921) and by comparative cognition research for close to half a century (McGonigle and Chalmers 1977). However, the mechanisms underlying transitive inference are not fully understood (Holyoak and Lu 2021).

One area of ongoing research in the study of transitive inference is the role of time- and sleep-dependent consolidation in the formation and retention of transitive inference abilities. Several studies have proposed that the process of memory consolidation, particularly that which occurs during sleep, plays an integral role in this cognitive phenomenon. This is based on the premise that sleep serves not only to stabilize memories but to qualitatively transform them, and this transformation may result in the abstraction of the gist of a memory or the discovery of latent structures underlying a learned material (Lewis and Durrant 2011; Inostroza and Born 2013; Lewis et al. 2018; Lerner and Gluck 2019). In one of the first studies investigating the impact of time and sleep on performance in the transitive inference task, Ellenbogen et al. (2007) reported that participants performed transitive inferences significantly above chance if they were allowed a night of sleep between training and testing, whereas those tested immediately after the session were at chance. This study also found that there was a more pronounced improvement in performance on more distant inference pairs (e.g., B?E, which requires two inferential steps to determine their relationship: C and D) when participants had slept between the training and testing session. The symbolic distance effect (SDE) is a phenomenon observed in various cognitive psychology experiments where reaction times and/or accuracy are affected by the numerical or conceptual distance between stimuli (Moyer and Landauer 1967). Specifically, response times decrease and accuracy increases as the distance between stimuli increases. It has been observed in various tasks and, in the context of transitive inference, has been used to support the idea that participants learn the relative ranks of stimuli in the hierarchy. While Werchan and Gómez (2013, 2016) partially support this sleep-dependent symbolic distance effect, their amended results are not technically significant, and other variations also failed to replicate the effect. For instance, a shorter delayed test (3 h) involving either a short nap or no nap (Morgan and Stickgold 2017) and within-subject studies involving a 12-h delayed test on a six-item hierarchy (Matorina and Poppenk 2021) or 24-h delayed test on a seven-item hierarchy (Berens and Bird 2022) found above-chance inference performance at delayed test but no time-dependent SDE.

Studies have suggested that time and, more specifically, sleep play a crucial role in the consolidation of newly acquired relational knowledge, potentially helping to strengthen the connections between stimuli in a hierarchy and improve overall inference performance (Lau et al. 2010; Alger and Payne 2016). Others have argued that many earlier findings showing sleep-mediated benefits on memory are not robust (Cordi and Rasch 2021) and that sleep-dependent benefits in statistical learning tasks are highly task-dependent and implicit only (Lerner and Gluck 2019). We propose that using diverse study designs and control variables can facilitate the elucidation of boundary conditions for time- and sleep-dependent gist extraction. Although considerable knowledge exists regarding moderators of sleep-related benefits for nonrelational episodic memories (Berres and Erdfelder 2021), it remains unclear how these factors pertain to sleep-dependent generalization studies (Pereira et al. 2023). An essential factor of interest is encoding strength, as studies may establish various learning criteria in their study phase, depending on whether previous research suggested that sleep enhances the retention of weaker memories to a greater extent than stronger memories (Diekelmann et al. 2009) or vice versa (Tucker and Fishbein 2008). While the use of a learning criterion can ensure that participants have acquired a minimum level of proficiency, individual differences in acquired performance can lead to varying sleep benefits (Denis et al. 2021). We hypothesize that encoding strength will affect not only overall inference performance but also qualitative measures of hierarchical learning. Thus, we not only expect to replicate earlier findings showing sleep-dependent differences in symbolic distance effect (a measure of relative positional encoding) but also propose an additional measure of absolute positional encoding based on the summed rank of a given pair's constituent item ranks (e.g., if A > B > C the summed rank of A?B would be 1 + 2 = 3, and for B?C it would be 2 + 3 = 5), referred to here as joint rank value (Jensen et al. 2017).

This is motivated by two strands of research. On the one hand, a study by Kao et al. (2020) extended the single-hierarchy transitive inference (TI) paradigm by using a derived list (or derived hierarchy) approach, in which participants were instructed to learn the ordinal structure of five hierarchies consisting of five items each (e.g., A_H1_ > B_H1_ C_H1_ > D_H1_ > E_H1_, …, A_H5_ > B_H5_ > C_H5_ > D_H5_ > E_H5_). Participants were then tested on both adjacent and nonadjacent pairs of items from five different derived hierarchies, and responses were scored such that the hierarchies were mixed but the ordinal positions of all items on the derived hierarchy were maintained. This means that the positions of items that were learned during training sessions retained their ordinal positions during testing sessions but were paired with novel items from different hierarchies (e.g., A_H1_ > B_H3_ > C_H5_ > D_H2_ > E_H3_, …, A_H5_ > B_H4_ > C_H2_ > D_H3_ > E_H1_). The investigators found evidence of greater than chance accuracy on these novel pairings during test as well as an SDE and argued that it is only possible for transitive inference to emerge in derived hierarchies if paired with an additional representation of absolute position.

On the other hand, Ciranka et al. (2022) focused on the behavioral modeling of emergent transitive inference during relational learning. They showed that inference performance is worse for inference trials with higher compared with lower values for joint rank [e.g., accuracy(B?D) ≪ accuracy(C?E)]. The investigators argued that this observed reduction in the ability to differentiate between the more dominant items (e.g., A?B, pair with low joint rank value) compared with the less dominant items (e.g., E?F, pair with high joint rank value) could be caused by compressed representations of magnitude that can emerge from an asymmetric learning policy. In other words, if participants consistently update their belief only about the winner (or loser) during premise pair learning, the model predicts diminishing rank-based discrimination as a function of joint rank value.

In two experiments, we studied the effect of encoding strength, measured by immediate testing performance, on sleep (experiment 1, Fig. 1A) and time-dependent (experiment 2, Fig. 1B) generalization while also examining the distance effect (Fig. 2A) and joint rank effect (Fig. 2C) and how these interact with consolidation across a period that includes sleep.

**Figure 1.**
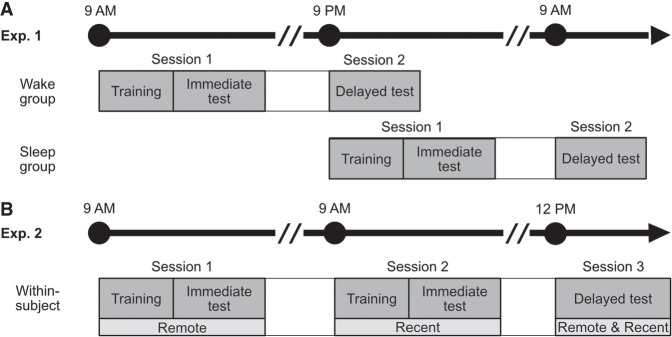
Experimental procedure. (*A*) Experiment 1. Participants were randomly assigned to either the wake group or the sleep group. The wake group started the experiment at 9:00 a.m. (±30 min), and the sleep group started the experiment at 9:00 p.m. (±30 min). Both groups followed the same procedure. In session 1, participants learned three separate hierarchies to criterion (premise pairs only). Immediately after training, participants were tested on all three hierarchies (premise pairs only). In session 2, 12 h later, participants were tested again on all three hierarchies, as well as on the novel inference pairs. (*B*) Experiment 2. All participants started between 9:00 a.m. and 11:00 a.m. In session 1, participants learned one hierarchy (remote; premise pairs only). Immediately after training, they were tested on these premise pairs. In session 2, 24 h later, participants learned a second hierarchy (recent; premise pairs only). Immediately after training, they were tested on these new premise pairs. In session 3, 3 h later, participants were tested again, this time on premise pairs from both remote and recent hierarchies, as well as their respective novel inference pairs.

**Figure 2.**
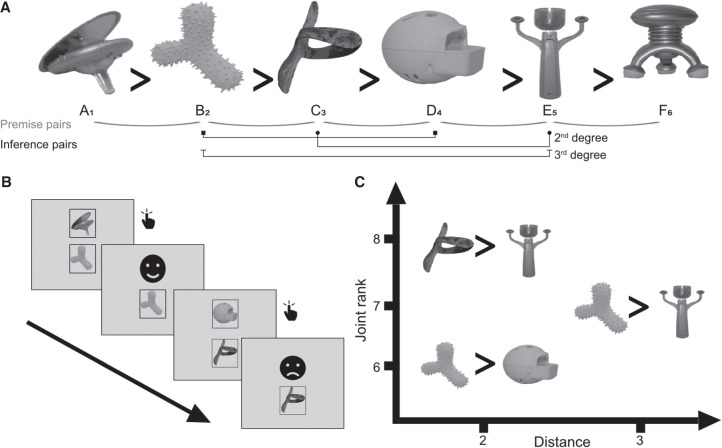
Experimental design. (*A*) Example hierarchy and hidden relational rank-order structure. Participants were presented with randomly generated hierarchies from a stimulus set that involved either faces, scenes, or objects. Adjacent premise pairs (e.g., A?B) were used during training, and nonadjacent inference pairs were used during a delayed test to assess relational learning. (*B*) Example training trials. Participants are asked on each trial to select the item hiding a smiley face and were given feedback after each selection. (*C*) Stimulus pairs can be represented along two orthogonal feature dimensions: symbolic distance (the difference in rank), which is represented on the *X*-axis, and joint rank (the sum of the ranks), which is represented on the *Y*-axis.

## Results

### Experiment 1

#### Behavioral results

**Figure 3.**
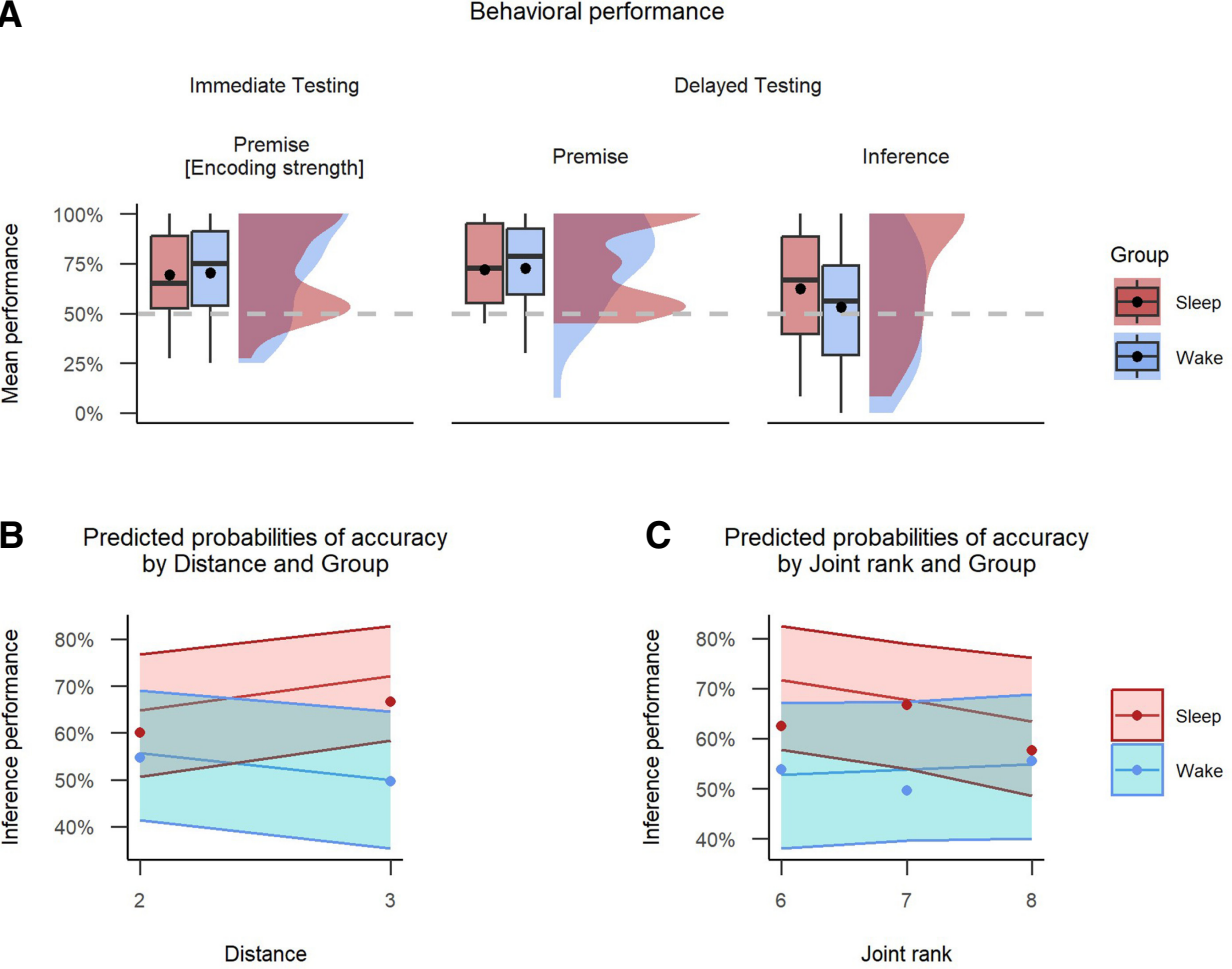
Experiment 1. Behavioral performance and factors predicting inference accuracy at delayed test. (*A*) Rain cloud plot with mean and median performance across wake and sleep conditions. The dashed line represents chance performance at 50%, and the dot represents mean values. (*B*) Predicted probabilities of accuracy by distance and group, with distance levels of 2 and 3. (*C*) Predicted probabilities of accuracy by joint rank and group, with joint rank levels ranging from 6 to 8. In *B* and *C*, shadowed areas represent 95% confidence intervals.

Our primary interest was the inference pairs, which were tested only at session 2 (postretention interval). One-sample *t*-tests showed that inference performance was significantly higher than chance for the sleep group (*M* = 0.62, 95% CI [0.54, ∞], *t*_(35)_ = 2.53, *P* = 0.008) but not for the wake group (*M* = 0.53, 95% CI [0.45, ∞], *t*_(35)_ = 0.62, *P* = 0.271) (Fig. 3A). These results suggest a sleep-dependent benefit to TI.

To test for baseline differences in premise pair memory that might have confounded the above result, we performed a mixed three-way ANOVA on the mean premise pair accuracy measure with between-subjects factor group and within-subject factor session and stimulus category. This revealed no effect for either of the factors (smallest *P* = 0.088) or their interactions (smallest *P* = 0.733) (Supplemental Table S2; Supplemental Fig. S1A). Note that inference pairs were not tested at baseline. For detailed descriptive statistics, see Supplemental Table S1.

#### Encoding strength

To assess the effects of our factors of interest (encoding strength and group) on inference performance at test, we next conducted a hierarchical regression with a series of nested mixed-effects logistic models. These models were random intercept-only models, with “participant” serving as the random intercept. This design allowed us to account for individual variations among participants. We started with the group variable, which captured whether the participant was in the sleep or wake condition; however, this did not outperform the intercept-only null model [χ^2^(1) = 1.84, *P* = 0.18]. We then added the encoding strength variable, which measured the mean accuracy of premise pair recall at immediate testing. This significantly improved the model's ability to predict accuracy compared with the null model [χ^2^(2) = 7.92, *P* = 0.019]. Next, we added an interaction term between group and encoding strength, which again significantly improved fit [χ^2^(1) = 15.15, *P* < 0.001]. In this final model, there was an interaction between group and encoding strength ($\hat{\text{β}}$ = −0.31, 95% CI [−0.47, −0.15], *z* = −3.89, *P* < 0.001) (Table 1), suggesting that the effect of encoding strength on accuracy differs significantly between the wake and sleep groups. To probe the interaction, simple effects coefficients were computed at 60% (OR_sleep/wake_ = 1.30, SE = 1.52, *P* = 0.53) and 80% (OR_sleep/wake_ = 2.42, SE = 1.52, *P* = 0.03) values of encoding strength, suggesting increased sleep-dependent benefit with stronger encoding.

**Table 1.**
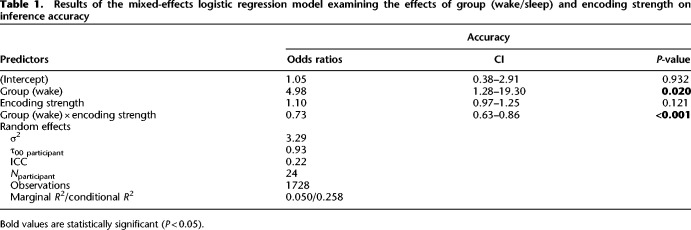
Results of the mixed-effects logistic regression model examining the effects of group (wake/sleep) and encoding strength on inference accuracy

#### Distance

To evaluate the influence of distance on inference performance at test, we continued the hierarchical regression analysis, extending previous findings of the “group and encoding strength interaction” model described above (Table 1, baseline model). We first added the distance variable, which captured whether a given trial was a “distant” (B?E; degree of separation: 3) or “close” (B?D or C?E; degree of separation: 2) inference trial. This did not significantly improve the fit model [χ^2^(1) = 0.11, *P* = 0.74]. Next, we added an interaction between group and distance, which significantly improved fit [χ^2^(1) = 6.41, *P* = 0.011]. Finally, we included a full factorial combination of the predictor variables group, encoding strength, and distance (comprised of all two-way interactions and a three-way interaction) (see the Materials and Methods). The additional terms did not significantly improve the model [χ^2^(2) = 3.93, *P* = 0.14]. The best-fitting model showed the same effects of group and encoding strength as reported by the baseline model. Additionally there was a main effect of distance ($\hat{\text{β}}$ = 0.34, 95% CI [0.02, 0.66], *z* = 2.07, *P* = 0.039) and a distance × group interaction ($\hat{\text{β}}\;$= −0.57, 95% CI [−1.01, −0.13], *z* = −2.53, *P* = 0.011) (Table 2). To investigate the interaction, we calculated the coefficients of simple effects at close (OR_sleep/wake_ = 1.47, SE = 1.52, *P* = 0.36) and distant (OR_sleep/wake_ = 2.59, SE = 1.55, *P* = 0.03) pair values of distance, suggesting increased sleep-dependent benefit for accuracy on inference pairs of greater symbolic distance. In summary, this analysis showed that the symbolic distance over which participants had to make an inference was a significant predictor of inference performance and also that the impact of this differed between sleep and wake groups, whereby the sleep benefit was especially evident for distant pair values (Fig. 3B).

**Table 2.**
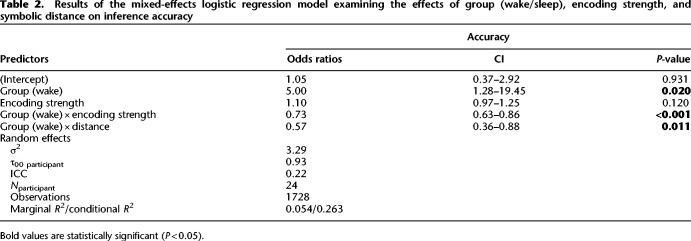
Results of the mixed-effects logistic regression model examining the effects of group (wake/sleep), encoding strength, and symbolic distance on inference accuracy

#### Joint rank

In parallel to the above, we also assessed the effects of joint rank on inference performance at test. We again started with our baseline model, first adding the joint rank variable, which encoded the summed rank of the inference pairs (B?D: 6, B?E: 7, and C?E: 8). This did not significantly improve the model [χ^2^(1) = 0.34, *P* = 0.56]. We next added the interaction between group and joint rank, which again did not significantly improve the model [χ^2^(2) = 1.94, *P* = 0.38]. Finally, we included a full factorial combination of the predictor variables group, encoding strength, and joint rank. The additional terms significantly improved the fit [χ^2^(4) = 12.14, *P* = 0.016]. This model showed the same effects of group and group × encoding strength as the baseline model. In addition, it showed an effect of joint rank ($\hat{\text{β}}$ = 0.85, 95% CI [0.21, 1.50], *z* = 2.59, *P* = 0.010), encoding strength × joint rank interaction ($\hat{\text{β}}$ = −0.15, 95% CI [−0.24, −0.05], *z* = −3.09, *P* = 0.002), and a three-way interaction between them ($\hat{\text{β}}$ = 0.12, 95% CI [0.00, 0.24], *z* = 1.99, *P* = 0.047) (Table 3). In order to examine the interaction, we computed the coefficients of simple effects for BD (JR: 6, OR_sleep/wake_ = 2.26, SE = 1.55, *P* = 0.06) and CE (JR: 8, OR_sleep/wake_ = 1.43, SE = 1.55, *P* = 0.41) trials. From this post-hoc analysis, we can conclude that for BD trials, the odds of success in the sleep group compared with the wake group were estimated to be 2.26 times higher, although this difference was not statistically significant (*P* = 0.06). In summary, this analysis showed that joint rank was a significant predictor and that it interacted with both baseline encoding strength and whether participants consolidated across sleep (Fig. 3C).

**Table 3.**
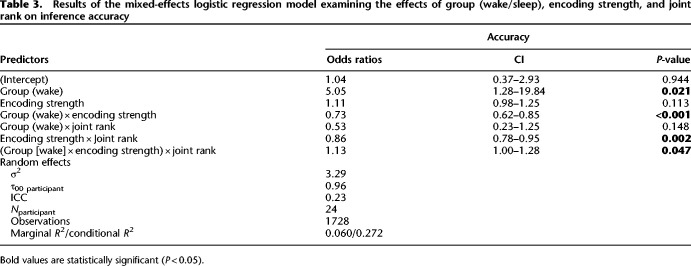
Results of the mixed-effects logistic regression model examining the effects of group (wake/sleep), encoding strength, and joint rank on inference accuracy

### Experiment 2

#### Behavioral results

**Figure 4.**
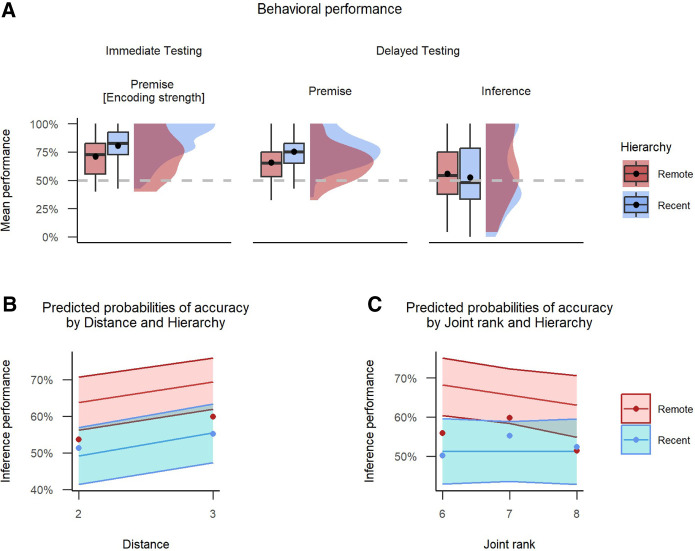
Experiment 2. Behavioral performance and factors predicting inference accuracy at delayed test. (*A*) Rain cloud plot with mean and median performance across remote and recent hierarchy conditions. The dashed line represents chance performance at 50%, and the dot represents mean values. (*B*) Predicted probabilities of accuracy by distance and hierarchy, with distance levels of 2 and 3. (*C*) Predicted probabilities of accuracy by joint rank and hierarchy, with joint rank levels ranging from 6 to 8. In *B* and *C*, shadowed areas represent 95% confidence intervals.

As in experiment 1, mean inference performance was significantly higher than chance for the remote hierarchy (*M* = 0.56, 95% CI [0.50, ∞], *t*_(69)_ = 1.80, *P* = 0.038) but not for the recent hierarchy (*M* = 0.53, 95% CI [0.47, ∞], *t*_(69)_ = 0.75, *P* = 0.228) (Fig. 4A). These results suggest a time-dependent benefit to TI.

To test for between-hierarchy baseline differences in premise pair learning, we performed a mixed three-way ANOVA on mean premise pair performance with within-subject factors hierarchy and session and between-subjects factor stimulus category. This revealed an effect of hierarchy (*F*_(1,67)_ = 33.61, *P* < 0.001, ${\hat{\eta }}_{G}^{2}=0.098$, 90% CI [0.015, 0.223]) with higher performance for the recent hierarchy, and session (*F*_(1,67)_ = 22.33, *P* < 0.001, ${\hat{\eta }}_{G}^{2}=0.027$, 90% CI [0.000, 0.120]) showing decreased performance at delayed test. There was no main effect of the stimulus category or interactions (smallest *P*-value = 0.32) (Supplemental Table S4; Supplemental Fig. S1B). Note that inference pairs were not tested at baseline. For detailed descriptive statistics, see Supplemental Table S3.

These findings suggest that for premise pairs there is a baseline difference in performance between the two hierarchies, whereby participants had higher accuracy for the recent hierarchy at both immediate and delayed tests (Fig. 4A).

#### Encoding strength

We examined the influence of encoding strength similar to experiment 1. We first added the hierarchy variable to the null model, which captured whether the participants’ performances relate to the remote or recent hierarchy. The addition of this variable significantly improved the model's ability to predict accuracy [χ^2^(1) = 4.00, *P* = 0.045]. Next, we added the encoding strength variable, which significantly further improved fit [χ^2^(1) = 107.39, *P* < 0.001]. Finally, we added an interaction term between hierarchy and encoding strength, which also significantly improved the model's ability to predict accuracy beyond the previous model [χ^2^(1) = 24.93, *P* < 0.001]. The results of this analysis suggest that both hierarchy and encoding strength have significant effects on accuracy and that their interaction also plays an important role in predicting accuracy on inference trials. As can be seen in Table 4, hierarchy was a significant predictor of the outcome variable ($\hat{\text{β}}$ = 1.54, 95% CI [0.68, 2.39], *z* = 3.51, *P* < 0.001), as was encoding strength ($\hat{\text{β}}$ = 0.58, 95% CI [0.47, 0.68], *z* = 10.92, *P* < 0.001) and the interaction between the two ($\hat{\text{β}}$ = -0.28, 95% CI [−0.39, −0.17], *z* = −4.98, *P* < 0.001). The interaction indicates that the effect of encoding strength on accuracy differs significantly between the remote and recent conditions. To probe the interaction, simple effects coefficients were computed at 60% (OR_remote/recent_ = 1.16, SE = 1.14, *P* = 0.24) and 80% (OR_remote/recent_ = 2.04, SE = 1.10, *P* < 0.001) values of encoding strength, suggesting an increased time-dependent benefit with stronger encoding.

**Table 4.**
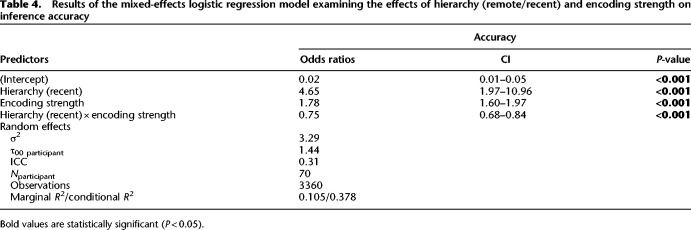
Results of the mixed-effects logistic regression model examining the effects of hierarchy (remote/recent) and encoding strength on inference accuracy

#### Distance

In order to assess how distance affects inference performance during testing, we conducted a hierarchical regression analysis that builds on the baseline model (Table 4). We first added the distance variable, which significantly improved the model's ability to predict accuracy compared with the baseline model [χ^2^(1) = 9.55, *P* = 0.002]. Next, we added an interaction term between hierarchy and distance, which did not significantly improve the model's ability to predict accuracy [χ^2^(1) = 0.51, *P* = 0.47]. Finally, we included the full factorial model (see the Materials and Methods), but the additional terms did not significantly improve the fit [χ^2^(3) = 2.47, *P* = 0.48]. The best fitting model showed the same effects of hierarchy, encoding strength, and hierarchy × encoding strength as reported by the baseline model. Additionally, there was a main effect of distance ($\hat{\text{β}}$ = 0.25, 95% CI [0.09, 0.41], *z* = 3.09, *P* = 0.002) (Table 5). In summary, this analysis showed that the symbolic distance over which participants had to make an inference was a significant predictor of inference performance, but this did not significantly differ between hierarchies (Fig. 4B).

**Table 5.**
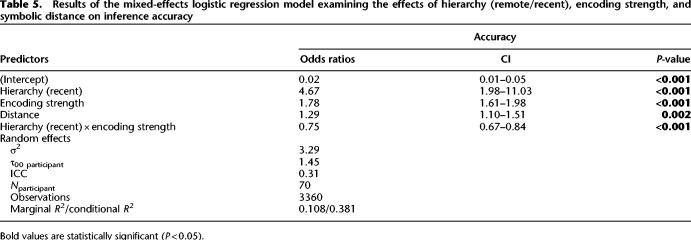
Results of the mixed-effects logistic regression model examining the effects of hierarchy (remote/recent), encoding strength, and symbolic distance on inference accuracy

#### Joint rank

In parallel, we assessed the effects of joint rank on inference performance at test. We first added the joint rank to the baseline model (Table 4), which did not significantly improve fit [χ^2^(1) = 0.38, *P* = 0.54]. Next, we added an interaction term between hierarchy and joint rank, which again did not significantly improve the model's ability to predict accuracy [χ^2^(2) = 3.47, *P* = 0.18]. Last, we compared a full factorial combination of the predictor variables hierarchy, encoding strength, and joint rank. The additional terms significantly improved the model's ability to predict accuracy [χ^2^(4) = 14.53, *P* = 0.0058]. The best fitting model showed the main effect for joint rank (), in addition to the effects shown in the baseline model. Furthermore, there was a two-way interaction effect between hierarchy and joint rank ($\hat{\text{β}}$ = −0.01, 95% CI [−1.93, −0.10], *z* = −2.17, *P* = 0.030) and a three-way interaction between hierarchy, encoding strength, and joint rank ($\hat{\text{β}}$ = −0.15, 95% CI [0.03, 0.27], *z* = 2.44, *P* = 0.015) (see Table 6). In order to examine the interaction, we computed the coefficients of simple effects for BD (OR_remote/recent_ = 2.03, SE = 1.14, *P* < 0.001) and CE (OR_remote/recent_ = 1.63, SE = 1.14, *P* < 0.001) trials. From this post-hoc analysis, we can conclude that for BD trials, the odds of success in the remote compared with the recent condition were estimated to be 2.03 times higher, while for CE trials it was only 1.63 times higher (Fig. 4C).

**Table 6.**
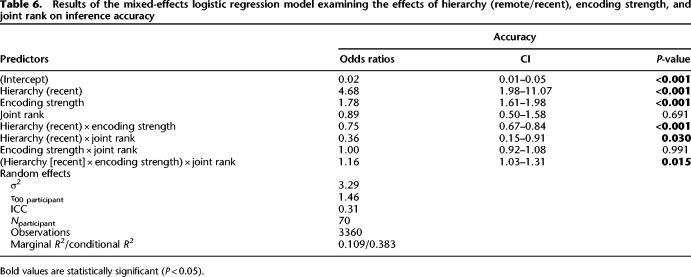
Results of the mixed-effects logistic regression model examining the effects of hierarchy (remote/recent), encoding strength, and joint rank on inference accuracy

## Discussion

The transitive inference (TI) paradigm evaluates how well individuals can grasp the relational arrangement of a group of stimuli without relying on any overt hierarchical cue while learning about them. To achieve this, the training involves displaying pairs of images selected from a predetermined list of neighboring items in order of rank and providing incentives for participants to correctly identify the dominant item. Work in both humans and other animals has supported the idea that the ability to assess relational dominance for nonneighboring inference items relies on some form of gist abstraction and that this is facilitated by time- and sleep-dependent memory consolidation in humans (Lewis and Durrant 2011; Inostroza and Born 2013; Lewis et al. 2018; Lerner and Gluck 2019). While initial findings (Ellenbogen et al. 2007; Werchan and Gómez 2013, 2016) showed strong time- and sleep-dependent benefits in inference performance and sleep-dependent SDE, subsequent studies that implemented modified parameters failed to replicate these findings, suggesting that the time/sleep–inference relationship may be more complex and dependent on specific experimental conditions (Cordi and Rasch 2021). Here, we evaluated whether we could partially replicate the original time- and sleep-dependent TI findings by Ellenbogen et al. (2007) in two experiments that involved learning multiple hierarchies with lower learning criterion and using a broader range of stimuli compared with the original study.

Crucially, our data support earlier findings (Ellenbogen et al. 2007; Werchan and Gómez 2013, 2016) by showing increased inference performance at delayed test in the experimental sleep group (experiment 1) (Table 1) and in the remote condition (experiment 2) (Table 4) compared with control. Interestingly, both of these effects increase as a function of encoding strength (Supplemental Fig. S2A,B). Our second experiment followed a design similar to that of Berens and Bird (2022), which found no time-dependent benefit in TI. We speculate that this could be due to differences in either difficulty (we used six items vs. their seven-item hierarchy) or encoding strength due to training duration (our 66% learning criterion vs. their large number of fixed trials for each hierarchy). While training to ceiling can eliminate baseline learning-related differences in premise pair encoding strength, which we observed in experiment 2 (Fig. 4), it can also exacerbate practice effects due to increased familiarity with task demands during the recent hierarchy condition, leading to altered consolidation dynamics (Denis et al. 2020, 2021; Petzka et al. 2021). In experiment 2, we observed a time-dependent benefit in TI despite weaker encoding strength in the remote compared with recent condition, even though we found encoding strength to be a strong predictor of delayed inference performance overall, which highlights the importance of retention interval × encoding strength interaction.

Concerning the sleep-dependent SDE shown in past work (Ellenbogen et al. 2007; Werchan and Gómez 2013), we were able to replicate this in our a.m.–p.m. design in experiment 1 (Fig. 3) but could not replicate it in the within-subject design of experiment 2. The latter results are a common pattern in studies using a longitudinal design (Matorina and Poppenk 2021; Berens and Bird 2022). This again could be caused by practice effects whereby SDE might take less time to emerge in the recent hierarchy condition due to familiarity with task demands.

To the best of our knowledge, we are the first to explore the time- and sleep-dependent joint rank effect (JRE). JRE—whereby participants, when comparing performance for pairs that have equivalent symbolic distance, exhibit lower accuracy the higher the cumulative sum of the item ranks—has been observed in both primates (Munoz et al. 2020; Ciranka et al. 2022) and humans (Jensen et al. 2017; Ciranka et al. 2022). JRE has been suggested as a valuable complementary variable to symbolic distance, as it serves as a measure of absolute positional encoding during serial learning and retrieval (Jensen et al. 2017) and is indicative of a cognitive model supporting TI (Behrens et al. 2018). The reduced ability to choose between more dominant items (e.g., B?D, pair with low joint rank value) compared with less dominant items (e.g., C?E, pair with higher joint rank value) observed in wake-only studies could be caused by compressed representations of magnitude that can emerge from an asymmetric learning policy (Ciranka et al. 2022). Interestingly, our two studies found that this pattern reversed across a retention interval containing sleep, with participants showing a lesser ability to discriminate during low-dominance inference pairs (C?E, JR: 8), and a greater ability to discriminate between high-dominance items (B?D, JR: 6) after consolidation (Figs. 3, 4). This “inverse joint rank effect” could suggest active time- and/or sleep-dependent consolidation processes, whereby experience is reorganized based on the learned absolute rank structure, prioritizing high-dominance items over low-dominance ones (van Rijn et al. 2016; Liu et al. 2019, 2021).

While the current study cannot provide a mechanistic explanation for this phenomenon, one explanation could relate to differences in wake/sleep replay dynamics, as it pertains to how veridical or “structured” online versus “disjointed” offline (in sleep) sequential reactivations can affect TI performance (Brodt et al. 2023). Moreover, while replay can be forward and reverse in both wake and sleep (and whether their relative frequencies differ across these states is still unclear), a dominance of forward replay in sleep could potentially explain our observation of inverse joint rank after consolidation across sleep (Findlay et al. 2020). A further explanation could involve offline replay prioritizing dominant, high-valued items at the expense of lower-ranked items (Momennejad 2020). Extending existing computational models of transitive inference with biologically inspired replay and fitting to delayed test data could help adjudicate between these possibilities (Mattar and Daw 2018; Jensen et al. 2019; Ciranka et al. 2021; Roscow et al. 2021).

One limitation of this study stemmed from the fact that we did not record sleep. Although experiment 1 compared consolidation across 12 h of wake and 12 h including an overnight sleep, we cannot assume that the benefit observed in the overnight condition relates specifically to sleep rather than to a combination of wake and sleep. Our second study suffered from the same problem but to an even greater extent, as we now compared consolidation across 27 h, including a night of sleep, versus consolidation across 3 h. Future studies could determine the specific importance of sleep for these effects more definitively by recording polysomnography.

A further limitation of our study was the use of a relatively small sample size in our first experiment. Although we were able to obtain significant results, a larger sample size would have increased the generalizability and robustness of our findings. Additionally, while we used well-established transitive inference tasks to assess memory consolidation, these tasks do not capture all aspects of relational memory, and other cognitive processes may have played a role in our results.

The results of our study have important implications for understanding the role of time and/or sleep in memory consolidation and suggest that both play a key role in the formation of the relational memories underlying transitive inference. Given that transitive inference is a fundamental cognitive process that is involved in a wide range of daily activities, our findings may have implications for developing computational models of consolidation and improving learning and memory in educational and clinical settings. Future studies may explore whether manipulating the online/offline replay or duration of sleep/wakeful rest can enhance transitive inference performance to better understand how rank-order effects emerge during learning and evolve over time, potentially leading to the development of interventions that can improve memory consolidation. Additionally, conducting sleep and memory studies using a derived hierarchies approach like the one used by Kao et al. (2020), where participants need to generalize across hierarchies, would provide a better chance at disentangling sleep-dependent effects on relative versus absolute encoding as well as investigating more flexible forms of generalization than single-hierarchy studies can.

In summary, we corroborated earlier findings showing that consolidation across a period of time including sleep benefits inference performance. We also showed that this benefit increases as a function of encoding strength and therefore argue that TI studies that use a “learn to criterion” approach during training should consider encoding strength as a relevant predictor, since there can be significant variation in premise pair performance using standard approaches. Our data, in combination with prior reports, suggest that the emergence of the sleep-dependent symbolic distance effect may be influenced by experimental design. Specifically, studies that use between-subjects designs have found such effects, whereas those using within-subject designs have not. The joint rank effect provides a complementary dimension of the mental model that participants used in solving this task, which is understudied in the transitive inference literature. We found that it is a strong predictor of inference performance and a sensitive measure of time- and sleep-dependent memory consolidation, affected by encoding strength.

## Materials and Methods

### Participants

#### Experiment 1

Twenty-four adults (age = 22 yr ± 3.72 yr) with no self-reported history of neurological, psychiatric, sleep, or motor disorders participated in the experiment. All participants provided written informed consent and were reimbursed for their time. The experiment was approved by the School of Psychology Ethics Committee at Cardiff University. All participants agreed to abstain from caffeine and alcohol during the study and for 24 h before it.

#### Experiment 2

A total of 74 participants completed the study, recruited from Prolific (https://www.prolific.co), an online platform for psychological research. Four participants were excluded from the study due to technical issues related to multiple submissions in either session 1 or session 2. Participants (age = 23.37 yr ± 4.1 yr) had normal or corrected to normal vision, with no self-reported history of neurological, psychiatric, sleep, or motor disorders. All participants provided informed consent electronically and were reimbursed for their time. The experiment was approved by the School of Psychology Ethics Committee at Cardiff University. All participants agreed to abstain from caffeine and alcohol during the study and for 24 h before it.

### Procedure

#### Experiment 1

Participants were randomly assigned to one of two groups: wake or sleep. Two participants were discarded for not being able to reach the criterion (66% accuracy in two consecutive blocks on middle pairs). Both groups participated in two sessions: an initial training with an immediate test session and a delayed test session separated by 12 h. Wake group participants arrived at the laboratory at 9:00 a.m. (±30 min) for the first session and came back at 9:00 p.m. (±30 min) for the second one, carrying on with their normal daily routines. Those in the sleep group arrived at the laboratory at 9:00 p.m. (±30 min) and came back the next morning at 9:00 a.m. (±30 min).

##### Premise pair training

Training involved the presentation and learning of the five-item pairs of each of the stimulus categories in a six-item hierarchy, referred to here as “premise pairs.” A hierarchy can be schematically represented with letters A > B > C > D > E > F where “>” describes the relationship “choose over” (e.g., “A > B” denotes “choose ‘A’ over ‘B’”). The order within the hierarchy was randomly selected for each participant at the start of the training phase. Premise pairs were presented on the screen, such that one image was located at the top of the screen and the other one was located at the bottom. On each trial, after the participant saw one of the five premise pairs (either A?B, B?C, C?D, D?E, or E?F), they were required to identify the correct item through a process of trial and error (Fig. 2B). However, with repeated exposure and feedback, participants were able to learn the correct item and make accurate selections. If the participant selected the correct item of the pair, the chosen item was replaced by a smiling face stimulus on the left side of the screen, and the other item was presented on the right side. When participants selected the wrong member of the pair, the chosen item was substituted by an angry face stimulus also on the left side of the screen and the other item was on the right. Finally, a purple circle in the middle of the screen was presented to indicate the end of each trial.

Items were organized into blocks, each containing 10 trials of each stimulus category (a total of 30 trials per block). Therefore, each block presented each of the five items of each hierarchy twice, counterbalancing the position from top to bottom or vice versa (e.g., A?B and B?A, where A was the correct selection in both trials). Additionally, all the premise pairs within each hierarchy were presented in a pseudorandom order to minimize the chance of revealing the latent hierarchy (e.g., A?B was never followed by B?C). At the end of each block, the mean accuracy for that block was shown on the screen to keep participants engaged with the task. Additionally, the order of the stimulus categories was counterbalanced across participants. All participants underwent a minimum of three blocks of training. After the third block, performance was automatically scored for each stimulus category. If the performance on the “middle premise pairs” (B?C, C?D, and D?E) for two of the last three blocks was >66% for any given hierarchy, the participant stopped receiving feedback for that particular hierarchy. Premise pairs were still displayed for such hierarchies to avoid different number of presentations across hierarchies. When the criterion, or a maximum of 10 blocks, was reached for all three stimulus categories’ participants, the program automatically stopped. Participants were given a 5-min break before advancing to the next phase.

##### Immediate and delayed test

During the immediate test, a block protocol similar to that, in the premise pair training was used with the exception that feedback cues were removed. Participants performed a total of four blocks, and in between blocks a series of two easy arithmetic problems had to be solved as a distractor task to clear the participant's short-term memory (von Hecker et al. 2019). Following a delay of 12 h, participants returned to the laboratory for the delayed test phase. This phase involved three novel inference pairs (B?D, B?E, and C?E) and an anchor pair (A?F), in addition to the five premise pairs. Participants were instructed that they may see novel combinations and, if that happened, to make their best guess on that trial. At the end of each trial, participants were asked how sure they were of their answer on a scale ranging from −2 (guessing) to +2 (completely sure). Similar to the immediate test, participants performed four blocks with two arithmetic problems between each block. After completing this phase, participants had to fill out a questionnaire to probe their awareness of the existence of a latent hierarchy underlying the items in each stimulus category. A mixed logistic regression analysis was conducted to analyze the data obtained from the immediate and delayed test phases, allowing for the examination of the effects of encoding strength, distance, and joint rank variables on participants’ performance.

#### Experiment 2

Participants were required to complete three sessions: the first session in the morning (between 9:00 a.m. and 11:00 a.m. local time), the second session 24 h later (between 9:00 a.m. and 11:00 a.m. local time), and the third session 3 h after completing the second session (or 27 h from the first). The first session had an initial training and immediate test part for only one hierarchy (named remote hierarchy). The second session was structured the same but participants learned a completely novel hierarchy (recent hierarchy). Finally, for the last session, a test involving both hierarchies was performed (delayed test).

##### Premise pair training

Training protocol was identical to experiment 1 with respect to instructions, stimuli, and learning criterion; the only difference was that participants learned only one hierarchy per training session. Participants had to reach the learning criterion (66% accuracy in two consecutive blocks on middle pairs) for a given hierarchy within 10 blocks.

##### Immediate and delayed test

The immediate test phase was also identical to experiment 1 and only tested the hierarchy learned in its respective session. Following a delay of 3 h after session 2, participants were required to complete the delayed test phase. Similar to experiment 1, this phase involved three novel inference pairs and an anchor pair, in addition to the five premise pairs for both remote and recent hierarchy, and lasted four blocks. Participants were instructed that they may see novel combinations and, if that happened, to make their best guess on that trial. Similar to the immediate test, participants performed four blocks with two arithmetic problems between them but were not asked to provide confidence ratings after each trial. After completing this phase, participants had to fill out a questionnaire to probe their explicit awareness of the existence of a latent hierarchy underlying the items for each hierarchy and, finally, complete a hierarchical recall test whereby they were asked to explicitly reconstruct the order of items in the hierarchy to assess implicit awareness. A mixed logistic regression analysis was conducted to analyze the data obtained from the immediate and delayed test phases, allowing for the examination of the effects of different variables on participants’ performance.

### Apparatus and stimuli

In experiment 1, a computerized memory task was presented in a quiet room using PsychToolbox (Kleiner et al. 2007), while in experiment 2 participants completed the experiment on their own computer or tablet device using a web browser (e.g., Chrome). Experiment 2 was programmed using PsychJS (Peirce 2007) and run through the Pavlovia platform (https://pavlovia.org). In the first experiment, the visual stimuli consisted of three sets of images (referred to here as stimulus categories), including female faces, unusual objects, and landscapes, each comprising six items. These items were selected randomly from a set of 12 images for each category. In the second experiment, participants were randomly assigned to one of the stimulus categories, with both the remote and recent hierarchies consisting of a six-item subset. All the items were presented in grayscale and matched for luminescence. Each item was distinguishable from the items within and between stimulus categories. In the first experiment, the order of the category presentation was counterbalanced across participants, and the order of the stimuli within each relational hierarchy was completely randomized for each subject in both experiments at the start of the learning phase.

### Statistical analyses

To examine the relative impact of encoding strength, distance, and joint rank on delayed inferential performance, separate hierarchical multiple regression analyses were conducted for distance and joint rank. It is crucial to highlight that these models were random intercept-only models, with “participant” serving as the random intercept. This design allowed us to account for individual variations among participants. Additionally, we used specific transformations on the variables used. First, we mean centered the distance and joint rank variables to enhance interpretability and reduce multicollinearity among predictor variables. Additionally, we applied a transformation to the encoding strength variable by multiplying the original values, which initially ranged between 0 and 1, by a factor of 10, enabling us to assess the impact of encoding strength in terms of a rate per unit of 10%.

To first identify the contributions of encoding strength, we entered our experiment-specific condition factor into the model in step 1 (group in experiment 1 and hierarchy in experiment 2), encoding strength in step 2, and the interaction of these measures at step 3 as predictors of memory recall for inferential pairs. Next, we extended the interaction model by adding in the distance factor as step 4, an interaction between distance and the respective condition factor as step 5, and finally a model that included a full factorial combination between condition, encoding strength, and distance as step 6. We also repeated this for joint rank replacing distance. An alpha value of *P* < 0.05 was used for all analyses.

In the regression tables, under the random effects header, various symbols represent different aspects of the statistical model. The residual variance (σ^2^) is a measure of the variability in the response variable “accuracy” that was not captured by the predictors included in the model. This served as an estimate of the average distance of each observation from the predicted values. The term τ_00_ participant denotes the variance of the random intercepts for “participants.” This means that we allowed each participant to have their own baseline “accuracy” value and estimated the variability of these baselines across all participants. The intraclass correlation coefficient (ICC) represents the proportion of total variability in “accuracy” that can be attributed to differences between participants. Finally, the marginal *R*^2^ and conditional *R*^2^ values provide estimates of how well the model explains the variability in “accuracy.” Marginal *R*^2^ represents the proportion of variance explained by the fixed effects alone, while conditional *R*^2^ takes into account both fixed and random effects (Nakagawa et al. 2017).

We used R (version 4.2.2) and the R packages lme4, afex, and emmeans for all our statistical analyses (Bates et al. 2015; https://CRAN.R-project.org/package=emmeans; https://CRAN.R-project.org/package=afex) and sjPlot for generating the regression tables (https://CRAN.R-project.org/package=sjPlot). We used the papaja package (https://github.com/crsh/papaja) to format and generate an APA-style documents.

## Supplementary Material

Supplement 1
