# Supplementary material for: Sleep-related benefits to transitive inference are modulated by encoding strength and joint rank
Source: Learn Mem. 2023 Sep;30(9):201–11. doi: 10.1101/lm.053787.123 (PMC10547378; doi:10.1101/lm.053787.123)
Supplement: Supplement 1 [file Supplemental_material.pdf]

Supplemental material

Supplemental Table S1: Descriptive statistics for Immediate and Delayed testing performance by Sleep and Wake groups. The table presents the median, mean, and standard deviation (SD) for performance during delayed testing, grouped by Sleep and Wake conditions. Data is further broken down by Pair values.

		**Group**					
		**Sleep**			**Wake**		
Session	Pair	Median	Mean	SD	Median	Mean	SD
Immediate testing	AB	0.88	0.63	0.42	1.00	0.70	0.41
Immediate testing	BC	0.81	0.69	0.34	0.81	0.62	0.42
Immediate testing	CD	0.69	0.69	0.31	0.88	0.70	0.33
Immediate testing	DE	0.75	0.71	0.30	0.88	0.71	0.34
Immediate testing	EF	0.88	0.75	0.31	1.00	0.78	0.37
Delayed testing	AB	0.94	0.67	0.40	1.00	0.73	0.39
Delayed testing	BC	0.88	0.75	0.34	0.88	0.67	0.41
Delayed testing	CD	0.88	0.73	0.30	0.81	0.71	0.34
Delayed testing	DE	0.88	0.71	0.36	0.88	0.72	0.34
Delayed testing	EF	0.88	0.74	0.34	1.00	0.81	0.34
Delayed testing	BD	0.62	0.62	0.35	0.62	0.54	0.39
Delayed testing	CE	0.62	0.58	0.38	0.62	0.56	0.42
Delayed testing	BE	0.75	0.67	0.35	0.50	0.50	0.37
Delayed testing	AF	0.88	0.69	0.39	1.00	0.70	0.40

Supplemental Table S2: Experiment 1: ANOVA table of baseline differences

Effect	$\hat{\eta}^2_G$	90% CI	F	df^{GG}	$df^{\mathrm{GG}}_{\mathrm{res}}$	p
Group	.000	[.000, .030]	0.01	1	22	.913
StimCategory	.020	[.000, .043]	1.53	1.95	42.82	.229
Session	.003	[.000, .118]	3.19	1	22	.088
Group × StimCategory	.002	[.000, .000]	0.16	1.95	42.82	.844
Group × Session	.000	[.000, .000]	0.01	1	22	.939
StimCategory × Session	.000	[.000, .000]	0.25	1.60	35.30	.733
Group × StimCategory × Session	.000	[.000, .000]	0.07	1.60	35.30	.902

Supplemental Table S3: Descriptive statistics for Immediate and Delayed testing performance by Remote and Recent condition. The table presents the median, mean, and standard deviation (SD) for performance during delayed testing, grouped by Remote and Recent conditions. Data is further broken down by Pair values.

		**Condition**					
		**Remote**			**Recent**		
Session	Pair	Median	Mean	SD	Median	Mean	SD
Immediate testing	AB	1.00	0.78	0.32	1.00	0.91	0.18
Immediate testing	BC	0.75	0.67	0.34	0.88	0.77	0.31
Immediate testing	CD	0.75	0.64	0.32	0.88	0.74	0.31
Immediate testing	DE	0.62	0.63	0.35	0.81	0.73	0.32
Immediate testing	EF	1.00	0.80	0.30	1.00	0.86	0.25
Delayed testing	AB	0.88	0.74	0.30	1.00	0.86	0.24
Delayed testing	BC	0.50	0.53	0.37	0.88	0.67	0.36
Delayed testing	CD	0.75	0.63	0.34	0.88	0.72	0.31
Delayed testing	DE	0.62	0.59	0.36	0.75	0.68	0.31
Delayed testing	EF	1.00	0.78	0.32	1.00	0.82	0.29
Delayed testing	BD	0.62	0.55	0.36	0.50	0.51	0.37
Delayed testing	CE	0.50	0.52	0.35	0.50	0.52	0.35
Delayed testing	BE	0.62	0.60	0.33	0.56	0.55	0.35
Delayed testing	AF	1.00	0.85	0.24	1.00	0.84	0.27

Supplemental Table S4: Experiment 2: ANOVA table of baseline differences

Effect	$\hat{\eta}^2_G$	90% CI	F	df^{GG}	df^{GG}_{res}	p
StimCategory	.012	[.000, .064]	0.61	2	67	.544
Hierarchy	.098	[.015, .223]	33.61	1	67	$< .001$
Session	.027	[.000, .120]	22.33	1	67	$< .001$
StimCategory × Hierarchy	.007	[.000, .047]	1.13	2	67	.329
StimCategory × Session	.001	[.000, .000]	0.36	2	67	.698
Hierarchy × Session	.000	[.000, .000]	0.03	1	67	.865
StimCategory × Hierarchy × Session	.002	[.000, .000]	0.97	2	67	.384

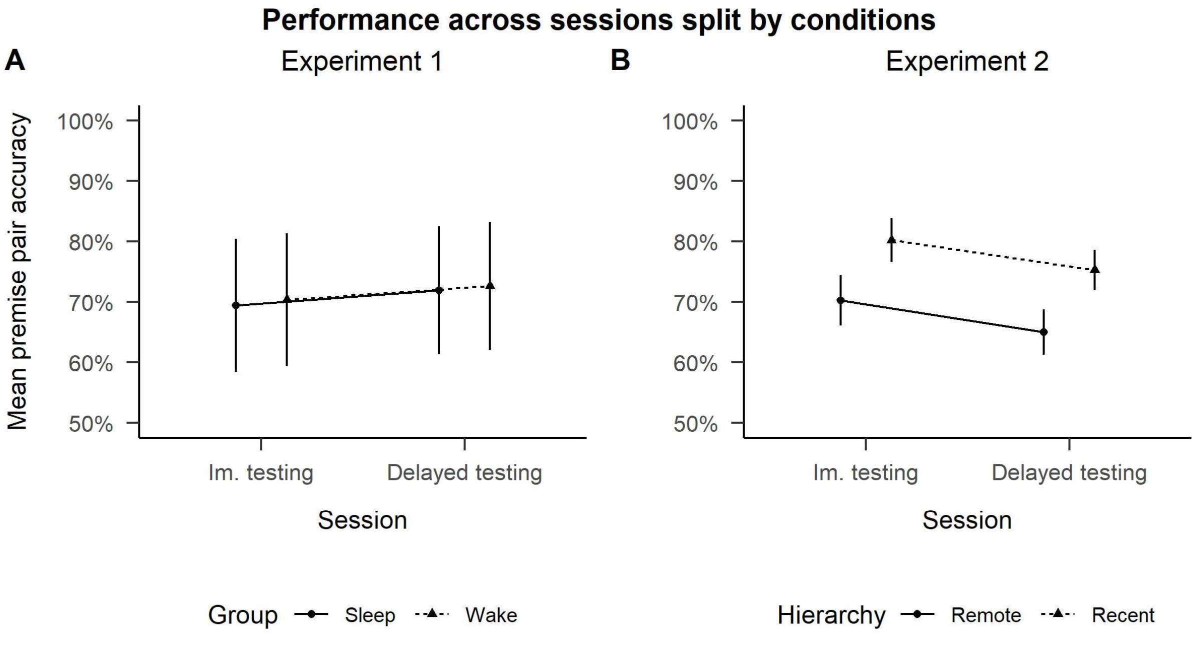

Supplemental Figure S1: A comparison of performance effects between conditions under varying delays. A) Mean premise pair accuracy for both the Sleep and Wake groups across sessions in Experiment 1. B) Mean premise pair accuracy for both Remote and Recent conditions across sessions in Experiment 2. The error bars represent the standard error of the mean.

a

Predictors	Odds Ratios	CI	p
(Intercept)	0.15	0.08 – 0.26	<0.001
Retention interval [Nosleep]	10.12	5.13 – 19.96	<0.001
Encoding strength	1.39	1.29 – 1.50	<0.001
Joint rank	1.35	0.88 – 2.07	0.164
Retention interval [Nosleep] * Encoding strength	0.70	0.64 – 0.77	<0.001
Retention interval [Nosleep] * Joint rank	0.55	0.30 – 1.01	0.052
Encoding strength * Jointrank	0.94	0.89 – 1.00	0.047
(Retention interval [Nosleep] * Encoding strength) * Joint rank	1.11	1.03 – 1.21	0.010
Random Effects			
σ2	3.29		
τ00participant	1.07		
ICC	0.25		
N participant	94		
Observations	5088		
Marginal R2 / Conditional R2	0.043 / 0.278		

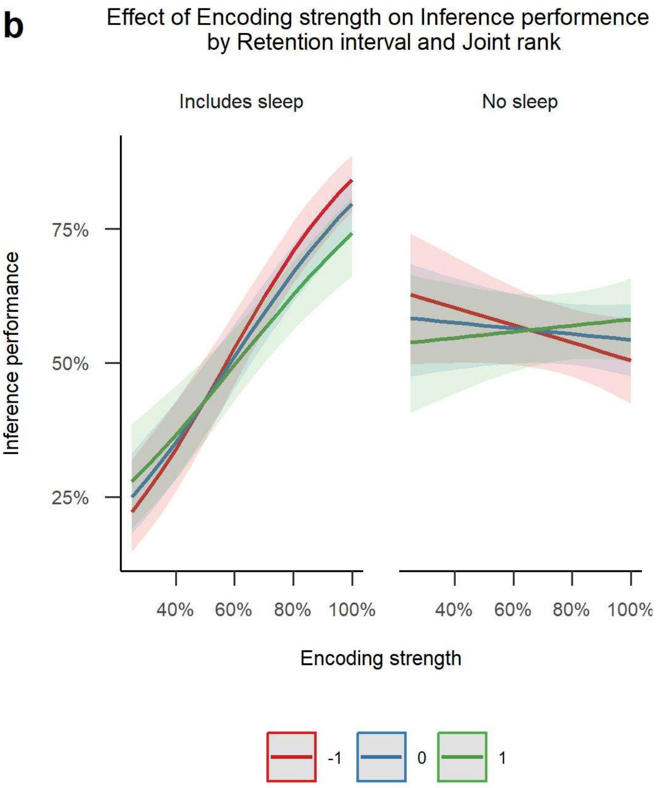

Supplemental Figure S2: Three-way interaction: Encoding strength X Joint rank X Retention interval A) Results of the mixed-effects logistic regression model examining the effects of Retention interval (Includes sleep vs. No sleep), Encoding strength, and Joint rank on inference accuracy. Data from Experiment 1 and Experiment 2 have been combined, with condition Sleep (Exp 1) and Remote (Exp 2) being relabeled as 'Includes sleep' and Wake (Exp 1) and Recent (Exp 2) as 'No sleep' B) Predicted probabilities of accuracy by Joint rank and Retention interval, with mean centered Joint rank levels ranging from -1 (B?D) to 1 (C?E). Shadowed areas represent 95% confidence intervals.
